# Predictors and incidence of sexually transmitted Hepatitis C virus infection in HIV positive men who have sex with men

**DOI:** 10.1186/s12879-017-2288-x

**Published:** 2017-03-02

**Authors:** Nicholas A. Medland, Eric P. F. Chow, Catriona S. Bradshaw, Timothy H. R. Read, Joseph J. Sasadeusz, Christopher K. Fairley

**Affiliations:** 1 Melbourne Sexual Health Centre, Alfred Health, Melbourne Australia and Central Clinical School, Monash University, Melbourne, Australia; 2 Royal Melbourne Hospital, Melbourne, Australia; 3 580 Swanston Street, Carlton, VIC 3053 Australia

**Keywords:** HIV, HCV, Sexual transmission, Sexually transmitted diseases, Syphilis, Chlamydia, Risk, Transmission, Men who have sex with men, Incidence, Coinfection

## Abstract

**Background:**

Sexual transmission of Hepatitis C virus (HCV) in men who have sex with men (MSM) and its interaction with HIV status, sexually transmitted infections and sexual behaviour is poorly understood. We assessed the incidence and predictors of HCV infection in HIV positive MSM.

**Methods:**

The electronic medical record and laboratory results from HIV positive MSM in care at a large urban public specialist HIV clinic embedded in a sexual health centre in Melbourne Australia were collected. Patients with two or more HCV antibody tests between January 2008 and March 2016 and with no record of injecting drug use were included. The HCV exposure intervals were the periods between a negative HCV test and the next HCV test. We compared HCV exposure intervals temporally associated with and without newly acquired syphilis or anorectal chlamydia. HCV exposure intervals were also categorised as being before or after HIV virological suppression and by most recent and nadir CD4 cell count.

**Results:**

Thirty seven new HCV infections were diagnosed in 822 HIV positive MSM with no history of injecting drug use over 3114 person years (PY) of follow-up. Mean age was 43.1 years (±12.5) and mean CD4 cell count nadir was 362 cells/uL (±186). The incidence of HCV infection in the study population was 1.19/100PY (0.99–1.38). The incidence in exposure periods temporally close to new syphilis infection was 4.72/100PY (3.35–6.08) and to new anorectal chlamydia infection was 1.37/100PY (0.81–1.93). The incidence in men without supressed viral load was 3.19/100PY (1.89–4.49). In the multivariate Cox regression analysis only younger age (aHR 0.67 (0.48–0.92)), exposure periods temporally associated to new syphilis infection (aHR 4.96 (2.46–9.99)) and higher CD4 cell count nadir (aHR 1.26 per 100 cells/uL (1.01–1.58)) were associated with increased risk of HCV infection. During the study period the incidence of syphilis increased dramatically but the incidence of HCV infection remained the same.

**Conclusions:**

Incidence of HCV infection is associated with syphilis but not anorectal chlamydia which suggests a biological rather than behavioural risk modification. Rising syphilis incidence may offset declines in HCV transmission through HCV treatment as prevention.

## Background

Sexual transmission of Hepatitis C virus (HCV), particularly amongst men who have sex with men (MSM), is now well established [1]. HIV-positive MSM are at highest risk, with seroprevalence up to 9% observed in this group [2, 3].

A number of factors have been associated with sexual, and presumed permucosal, transmission in MSM but it is difficult to separate out the relative contribution of each risk factor due to their interrelationship. Specifically, it is not clear how behavioural and biological factors influence transmission probability. Condomless receptive anal intercourse, practices that potentially expose the anorectal mucosa to blood (such as fisting, sex toys and douching) and a history of syphilis have all been associated with increased HCV risk amongst MSM [4–8]. Condomless receptive anal sex is also associated with an increased risk of anorectal chlamydia [9, 10]. However, anorectal chlamydia is not usually associated with genital ulceration [11, 12]. Syphilis is also associated with condomless receptive anal sex [13]. Unlike anorectal chlamydia, it is also associated with genital ulceration and is thus a marker of both behavioural and biological susceptibility. Similarly, how host factors related to HIV infection, like immunosuppression, viremia, antiretroviral therapy (ART), virological suppression and immune recovery, affect risk of sexual acquisition of HCV has not been studied.

The objective of this study was examine incidence of sexually transmitted HCV infection in a population of HIV positive MSM and to determine whether host biological factors, recent sexually transmitted infection (STI) associated with sexual behaviour but not with increased biological risk of infection (i.e. anorectal chlamydia) or a recent STI associated with both sexual behaviour and biological risk (i.e. syphilis) predicted it.

## Methods

We undertook a retrospective cohort study using the records of HIV-positive patients at the Melbourne Sexual Health Centre (MSHC) in the Australian state of Victoria. MSHC is a large publicly funded sexual health centre with an embedded specialist HIV clinic. Approximately 20% of the estimated 6300 individuals living with HIV in Victoria are current receiving care at MSHC [14].

MSHC uses a customised electronic medical record (Clinical Patient Management System, CPMS) which records detailed demographic, clinical and treatment history and results from investigations performed on site. CPMS contains all clinical data on all patients in care. The Melbourne Diagnostic Unit Public Health Laboratory (MDU PHL, University of Melbourne) provides onsite laboratory services including testing of microbiological specimens for *Neisseria gonorrhoeae* (Nucleic acid amplification testing (NAAT) and culture) and *Chlamydia trachomatis* (NAAT). The Victorian Infectious Diseases Reference Laboratory (VIDRL) is contracted to perform all off-site laboratory biochemistry testing including serology, virology and CD4 cell counts.

Data extracted from the electronic record included age, sex, country of birth, risk factor for HIV acquisition and results of anorectal chlamydia by NAAT. Anorectal chlamydia was chosen because it is associated with condomless receptive anal intercourse, which has also been associated with HCV infection, but not usually with a significant breach in the anorectal mucosa, i.e. ulceration, and because highly sensitive NAAT detection was used throughout the study period [9–12]. Gonorrhoea was not chosen because there was a change in detection method from culture to NAAT testing during the study period. Country of birth was defined as being within or outside Australia and New Zealand because of the large numbers of patients born in New Zealand and the similar HIV epidemiology in that country [15]. Data provided by the external laboratory included HIV viral load, CD4 cell count, HCV antibody and RNA testing, liver function tests and HBV serology for all HIV-positive patients at MSHC from January 1^st^ 2002 to March 31^st^ 2016. MSHC began annual screening for hepatitis C for all HIV positive patients in 2005.

Patients were included if they were male, in care at the MSHC HIV clinic, had two or more HCV antibody tests between January 1st 2008 and 31^st^ March 2016, their first HCV antibody test was negative, had sexual contact with men as their recorded risk factor for HIV acquisition and had no recorded history of injecting drug use (IDU). The clinical files of patients who were diagnosed with HCV infection during the study period were examined further and patients were excluded if their clinical file contained any report of injecting drug use, or use of blood products.

Diagnosis of HCV infection was made with either HCV antibody testing or, in some cases was initially made through HCV quantitative or qualitative DNA testing and followed up with antibody testing. HCV serology was performed using the Murex anti-HCV v4.0 ELISA assay with supplementary testing by Bio-Rad Monolisa anti-HCV-2 Plus EIA. HCV qualitative polymerase chain reaction (PCR) testing was performed using Roche Ampliprep/Cobas Taqman qualitative test version 2.0 and HCV viral load was performed using bDNA Bayer Version 3.0 in accordance with the Australian National Hepatitis C testing policy [16]. Syphilis serology was performed using Rapid Plasma Reagin (RPR) (Macro-Vue RPR card), Treponema pallidum Particle Agglutination assay (TPPA) (Serodia TPPA), a recombinant total antibody enzyme-linked immunosorbent assay (EIA) (Trepanostika TP recombinant; and ELISA immunoassay (EIA) Bio-Merieux). From January 2016 the Biomerieux EIA was replaced by with a LIASON Treponema screen (DiaSorin), an automated chemiluminescence immunoassay. Chlamydia NAAT testing was performed using the Becton Dickinson ProbeTec strand displacement amplification assay until March 2015 and then the Aptima Combo 2 (Hologic) transmission-mediated amplification assay.

Each HCV exposure interval was defined as the interval between a negative HCV antibody test and the following HCV antibody test. Each interval ended with either a negative HCV antibody test (censor) or with a positive HCV antibody test or HCV DNA PCR test (event). Only HCV exposure intervals which began on or after 01 January 2008 were included in the analysis. However, results from before 01 January 2008 were used to establish baseline measures for syphilis serology, CD4 cell counts, HIV viral load, HBV serology and STI testing.

We used HIV viral load as a marker of successful use of ART with virological suppression. HCV exposure intervals that began on or after the date of first recorded HIV viral load of less than 200 copies/ml were defined as occurring after virological suppression. For immune function, we used the most recent CD4 cell count recorded before the end of the HCV exposure interval. We also included the nadir CD4 count.

We defined incident syphilis as any seroconversion from non-reactive to reactive syphilis serology or, in the case of syphilis re-infection, greater than 4 fold increase in rapid plasma reagin (RPR) since most recent previous testing. We defined incident anorectal chlamydia as a positive or reactive *Chlamydia trachomatis* NAAT result in a patient whose most recent prior NAAT result was negative. The incident syphilis or anorectal chlamydia diagnostic interval was the period from the most recent negative test to the positive test.

**Fig. 1:**
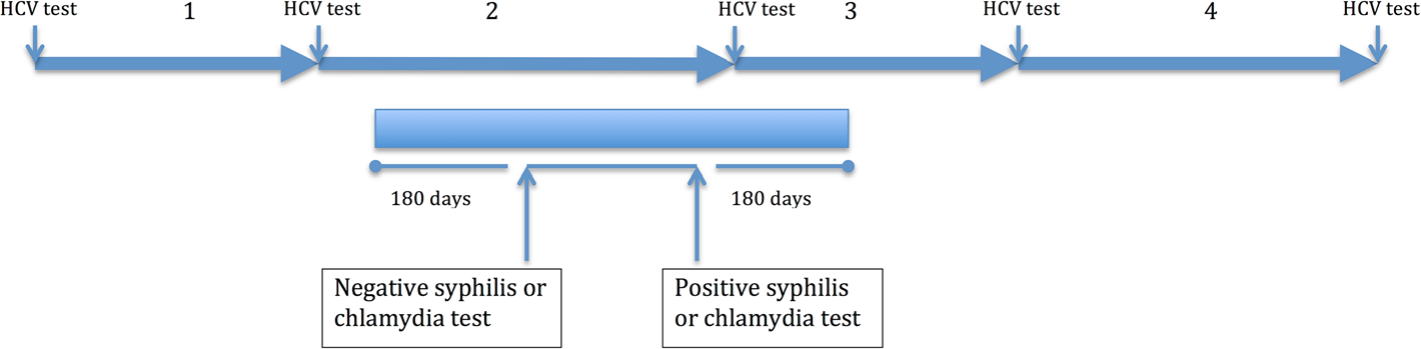
Definition of HCV exposure *peri*-incident syphilis or chlamydia. Four consecutive HCV exposure intervals in the same patient (numbered 1–4): 2, 3: These HCV exposure intervals are included as *peri*-incident syphilis or anorectal chlamydia: 1, 4: These HCV exposure intervals are not included as peri-incident syphilis or anorectal chlamydia. Notes: An HCV exposure interval is the period between a negative HCV antibody test and the following HCV antibody test. An HCV testing exposure period is *peri*-incident syphilis or *peri*-anorectal chlamydia if it overlaps with an interval extending from 180 days before to 180 days after the period between a negative syphilis or anorectal chlamydia test and an immediately following positive syphilis or anorectal chlamydia test

We defined the period of HCV follow up associated with either syphilis or chlamydia as *peri*-incident syphilis or *peri*-incident anorectal chlamydia. It was not possible to determine exactly when an individual acquired either syphilis or chlamydia except that it was within the period between a negative and positive tests. Furthermore the HCV seroconversion window could potentially be up to 180 days after either of these infections. To account for these two issues we conservatively assumed that it was possible for HCV infection to have occurred up to 6 months after a positive syphilis or chlamydia test or up to 6 months before a negative test (Fig. 1). Figure one illustrates this graphically.

Patients were defined as *ever HBV infected* if there was a positive HBV core antibody result recorded before or during the study period and *possible incident HBV* if they had a negative HBV core antibody result followed by a positive HBV core antibody result during the study period.

All analyses were conducted using the IBM SPSS Statistics software package (version 23). Univariate Cox proportional hazards regression analysis was performed on HCV testing intervals with the following covariates: age, country of birth, HCV exposure peri-incident syphilis, HCV exposure peri-incident anorectal chlamydia, HCV exposure after virological suppression, year of test, possible incident HBV infection and and ever HBV infected, as defined above. Multivariate Cox proportional hazards regression analysis was performed by adjusting for potential confounders. Covaraties with *p*-value < 0.20 in the univariate analysis were included in the multivariate analysis.

The study was approved by the Alfred Human Ethics Committee (approval number 189/16).

## Results

Eight hundred twenty-two MSM who attended for specialist care and were tested at least twice for HCV antibodies between January 1^st^ 2008 and 31^st^ March 2016 were included after excluding 172 women, 34 men with both IDU and MSM risk, 9 men with IDU risk only and 316 men with other modes of HIV transmission as their only recorded risk, and 95 men with no recorded risk factor. The mean age of these men was 43.1 years, 312 (38%) were born outside Australia or New Zealand and the mean CD4 cell count nadir was 362 cells/uL. 205 men (24.9%) were diagnosed with incident syphilis during the study period and 60 (7.3%) on two or more occasions. See Table 1. These 822 MSM contributed 3114 person years of follow up.

**Table 1:** Characteristics of 822 HIV-positive MSM patients during the study period

	Total^a^ (*N* = 822)
Age in years, mean (±SD)^b^	43.1 (±12.5)
Born outside Australia or New Zealand, n (%)^c^	312 (38.0%)
CD4 nadir cells/uL, mean (±SD)	362 (± 186)
Years since HIV diagnosis, mean (±SD)^b^	6.8 (±7.2)
Never supressed viral load, n(%)^d^	28 (3.4%)
Any incident syphilis, n(%)^e^	205 (24.9%)
2 or more syphilis, n(%)^e^	60 (7.3%)
Any anorectal chlamydia, n(%)^f^	165 (20.1%)
2 or more anorectal chlamydia, n(%)^f^	70 (8.5%)
Number HCV tests, mean(±SD)^g^	3.2 (2.0)
Ever HBV infected, n(%)^h^	232 (28.2%)
Possible incident HBV, n(%)^i^	5 (0.6%)
Patient years follow-up, mean (±SD)	3.8 (±1.6)

Notes

^a^All participants were MSM with no recorded history of injecting drug use

^b^On the day of the first HCV antibody test

^c^Place of birth outside Australia or New Zealand

^d^Viral load greater than 200 at every testing episode within the study period

^e^Incident syphilis defined as change from negative to positive specific syphilis serology or 4 fold increase in RPR during the study period

^f^Positive anal chlamydia NAAT test where the most recent previous negative test was negative during the study period

^g^Number of HCV antibody tests during the study period excluding the baseline negative test

^h^Any positive HBVcore antibody serology at any time during the study period

^i^Patients with positive HBV core antibody who previously had a negative HBV core antibody test during the study period

We identified thirty seven cases of incident HCV infection after exclusion of two further cases who reported injecting drug use at the time of HCV diagnosis. Thirty four of these were identified through HCV antibody testing, of whom 24 went on to have positive HCV DNA PCR. Three patients were initially diagnosed with HCV through a positive HCV DNA test and a negative antibody test. Of these 37 cases, 15 had grade three liver function test elevation at the time of diagnosis (greater than five times the upper limit of normal), ten had grade two (two to five times the upper limit of normal), three had liver function test elevations less than twice the upper limit or normal and nine had normal liver function tests.

The overall incidence of HCV infection in the study population was 1.19/100PY (0.99–1.38). The incidence in exposure periods temporally close to syphilis infection (*peri*-incident syphilis) was 4.72/100PY (3.35–6.08) and to anorectal chlamydia (*peri*-incident chlamydia) was 1.37/100PY (0.81–1.93). The incidence in men without supressed HIV viral load was 3.19/100PY (1.89–4.49). See Table 2.

**Table 2:** Incidence, crude and adjusted hazards ratio for incidence HCV infection. Cox Regression Analysis

	Incident HCV cases	PYs	Incidence (cases/100PY)	Unadjusted HR (95%CI)	*p*-value	Adjusted HR (95%CI)	*p*-value
Total	37	3114	1.19 (0.99–1.38)				
Age^a^	37	3193		**0.61 (0.45–0.83)**	**.002**	**0.67 (0.48–0.92)**	**.014**
Country of birth							
*Australia/New Zealand*	26	1927	1.35 (1.08–1.61)	1 (ref)			
*Other*	9	1053	0.85 (0.57–1.14)	0.64 (0.30–1.37)	0.250		
CD4 count ^b^							
*Most recent CD4*	37	3114		**1.11 (1.00–1.23)**	**.054**	1.00 (0.85–1.18)	.965
*Lowest CD4* ^*c*^	37	3114		**1.20 (0.95–1.52)**	**.121**	**1.26 (1.01–1.58)**	**.044**
Year of HCV Test	37	3114		1.12 (0.94–1.34)	.208		
HIV viral load suppression ^d^							
*No*	6	188	3.19 (1.89–4.49)	1 (ref)	–		
*Yes*	31	2926	1.06 (0.87–1.25)	**0.33 (0.14–0.79)**	**.012**	0.51 (0.20–1.27)	.146
Exposure *peri*-incident syphilis^e^							
*No*	25	2859	0.87 (0.70–1.05)	1 (ref)	-		
*Yes*	12	254	4.72 (3.35–6.08)	**5.73 (2.86–11.45)**	**<.001**	**4.96 (2.46–9.99)**	**<.001**
Exposure *peri*-incident anorectal chlamydia^f^							
*No*	31	2676	1.16 (0.95–1.93)	1 (ref)			
*Yes*	6	438	1.37 (0.81–1.93)	1.19 (0.49–2.84)	.703		
Ever HBV infected^g^							
*No*	29	2142	1.35 (1.10–1.61)	1 (ref)	-		
*Yes*	8	972	0.82 (0.53–1.11)	0.61 (0.28–1.33)	.214		

Notes

^a^Age at beginning of testing interval (HR for increments of 10 years)

^b^Most recent CD4 cell count before beginning of testing interval (HR for increments of 100cells/uL)

^c^Lowest ever recorded CD4 cell count (HR for increments of 100cells/uL)

^d^Most recent viral load before beginning of testing interval less than 200 copies/ml

^e^Any testing interval overlapping with a period 180 days before the beginning and 180 days after the end of the syphilis testing interval in which incident syphilis was diagnosed

^f^Any testing interval overlapping with a period 180 days before the beginning and 180 days after the end of the anorectal chlamydia testing interval in which incident anorectal chlamydia was diagnosed

^g^Any testing interval in any patient ever with positive HBV core antibody

**Fig. 2:**
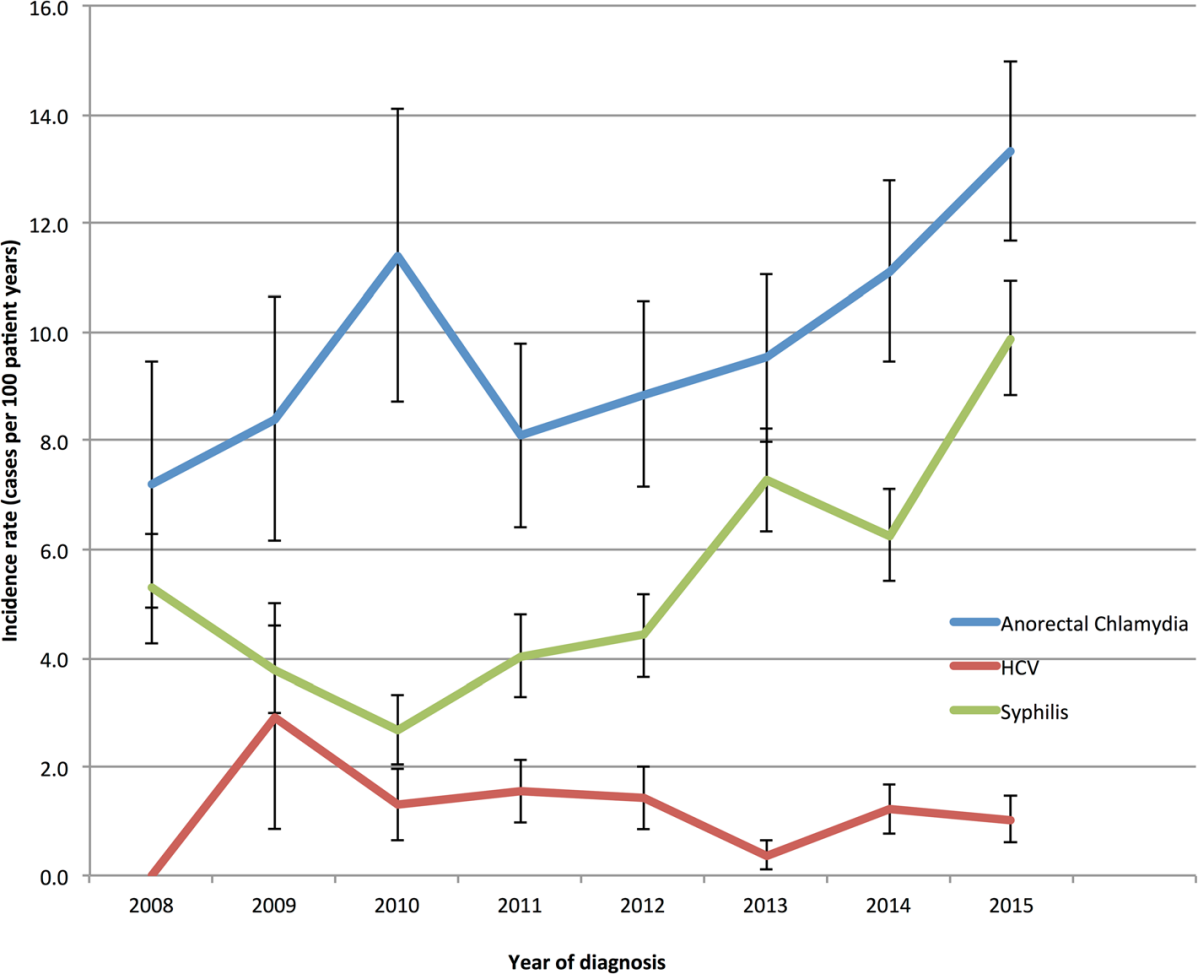
Incidence of HCV infection, Syphilis and Anorectal Chlamydia 2008 to 2015. Notes: Incidence was calculated only from testing intervals beginning after January 1 2008

The incidence of syphilis rose significantly from 5.3 /100PY in 2009 to 9.9/100PY in 2015(*p* = 0.035) and anorectal chlamydia rose significantly from 7.2/100PY to 13.3/100PY (*p* = 0.037) (See Table 3 and Fig. 2).

**Table 3:** Incidence of Anorectal chlamydia, syphilis and HCV infection 2008 to 2015

	Anorectal Chlamydia				Syphilis				HCV			
Year^a^	PY	No. of cases	Incidence, per 100 patient years (*p* = 0.037)^b^	Testing interval, Median, days (IQR)	PY	No. Of cases	Incidence, per 100 patient years (*p* = 0.035)^b^	Testing interval, Median, days (IQR)	PY	No. of cases	Incidence, per 100 patient years (*p* = 0.741)^b^	Testing interval, Median, days (IQR)
2008	139	10	7.2 (4.9–9.5)	143 (91–252)	511	27	5.3 (4.3–6.3)	77 (43–99)	5	0	-	
2009	167	14	8.4 (6.2–10.7)	152 (87–347)	580	22	3.8 (3.0–4.6)	77 (42–98)	68	2	2.9 (0.9–5.0)	179 (76–364)
2010	158	18	11.4 (8.7–14.1)	169 (106–322)	672	18	2.7 (2.0–3.3)	79 (49–99)	308	4	1.3 (0.6–1.9)	342 (186–423)
2011	284	23	8.1 (6.4–9.8)	114 (83–252)	718	29	4.0 (3.3–4.8)	84 (48–105)	449	7	1.6 (1.0–2.1)	325 (167–399)
2012	305	27	8.9 (7.2–10.6)	112 (78–187)	747	33	4.4 (3.7–5.2)	83 (46–112)	423	6	1.4 (0.8–2.0)	292 (144–393)
2013	399	38	9.5 (8.0–11.1)	98 (65–168)	810	59	7.3 (6.3–8.2)	83 (41–112)	526	2	0.4 (0.1–0.6)	336 (212–398)
2014	395	44	11.1 (9.5–12.8)	99 (55–151)	895	56	6.3 (5.4–7.1)	84 (43–119)	569	7	1.2 (0.8–1.7)	299 (179–392)
2015	480	64	13.3 (11.7–15.0)	97 (56–169)	880	87	9.9 (8.8–10.9)	87 (48–135)	579	6	1.0 (0.6–1.5)	292 (157–374)

Notes

Incidence was calculated only from testing periods beginning after January 1 2000

^a^ Year of the case diagnosis

^b^Linear regression for association between incidence rate and year

Five patients seroconverted to HBV core antibody during the study period. Of these, one later seroreverted to negative HBV core antibody status, two others had high HBV surface antibody titre that did not change before and after core antibody seroconversion and only one had a reactive HBV core IgM antibody result. None of these five patients were infected with HCV during the study period. 232 patients (28.2%) had had a reactive HBV core antibody test at some point before or during in the study period.

On the univariate regression analysis younger age, higher CD4 cell count, HCV exposure intervals which occurred before virologically suppression, and *peri-*incident syphilis were associated with an increased hazards ratio for HCV acquisition. On the multivariate analysis, only higher CD4 cell count nadir (but not most recent CD4 cell count), younger age and *peri*-incident syphilis were associated with increased risk of HCV infection. Being born outside Australia or New Zealand, the year of the test, incident anorectal chlamydia and HBV core antibody status were not associated with increased risk of HCV infection. (See Table 2).

## Discussion

In this study of HIV positive MSM with no recorded history of injecting drug use, incident HCV infection was associated with incident syphilis infection but not with incident anorectal chlamydia in the adjusted analysis. Hepatitis C incidence was also associated with younger age and higher CD4 cell count nadir. In the unadjusted analysis, HIV viral load suppression was associated with a reduced incidence of HCV . Furthermore, while the incidence of syphilis has risen substantially over this time period, the incidence of HCV, at least in our cohort, has not. The stable incidence of HCV with a rising incidence of syphilis suggests that other factors may be putting downward pressure on hepatitis C transmission such as greater use of HIV treatment with more HIV virally suppressed individuals.

Our study was able to examine two key sets of factors affecting the incidence of HCV infection. Firstly, we were able to analyse HCV exposure periods that occurred near the time of infection with syphilis and anorectal chlamydia. This potentially separates the biological risk associated with syphilis from the behavioural risk associated with both syphilis and anorectal chlamydia. Secondly, we were able to examine CD4 cell nadir, virological suppression and CD4 cell nadir as covariates in a post-HAART era cohort.

Risk factors for HCV acquisition can be studied in either a cohort or case control study design. Case control studies are unable to measure incidence or to determine temporal relationship between disease and risk factors. Our study is the second cohort to study the association between incident syphilis infection and HCV incidence but the first to study it in a cohort only of HIV positive MSM with no recorded history of injecting drug use. Furthermore it is the first cohort to study both incidence of HCV and incidence of syphilis and anorectal chlamydia. Many studies with weaker designs have examined associations between HCV acquisition, sexual behaviour and sexually transmitted infections [4, 17–20]. However, whether the risk is attributable to the sexual behaviour or the sexually transmitted infection which is associated with that behaviour remains an open question which we sought to address in this study. A systematic review of the risk factors for sexually transmitted HCV in HIV positive MSM published in 2015 found only four studies that presented adjusted estimates of risk [19–22]. Of these four studies, two were case control studies which showed association between HCV acquisition and sexual behaviour [19, 20]. The other two were cohort studies that did not specifically measure syphilis incidence. Both published from the Swiss HIV cohort study group, they showed association between HCV incidence, sexual behaviour and a past history of syphilis, but were not able to examine incident syphilis [22]. The Multicentre AIDS Cohort Study (MACS cohort) examined HCV incidence in both HIV positive MSM (but not excluding those with a history of injecting drug use) and in MSM who have never injected (but not excluding HIV negative MSM) and found an association between HCV risk and both high risk behaviour and recent syphilis in the former and in high risk behaviour but not recent syphilis in the latter [7]. Our study found that incident syphilis was associated with an increased risk of HCV infection but incident anorectal chlamydia was not. This suggests that biological factors are responsible for the increased risk, rather than behavioural. In addition it is biologically plausible that HCV transmission is facilitated by genital ulcer disease.

The association between age and the incidence of HCV is unclear. Both an increase and decrease in the incidence of HCV have been associated with increasing age in different cohort studies [7, 22]. The case control studies mentioned in the paragraph above controlled for age in selecting controls and therefore could not determine an age effect [19, 20]. One cohort study observed a lower incidence of HCV with increasing age, although this was not discussed further [22]. The MACS cohort showed an association between increasing age and reduced incidence of HCV in the full cohort but an association between increasing age and increasing incidence of HCV in HIV positive MSM (which did not exclude people with a history of injecting drug use) and in people who did not inject drugs (including HIV negative MSM) [7]. They noted more condomless receptive anal intercourse partners in younger MSM and in those taking antiretroviral therapy [7]. Our study, which only included HIV positive MSM who did not inject drugs, observed a reduced incidence of HCV with increasing age. Our analysis controlled for STIs which were markers of biological and behavioural risk and for HIV virological suppression. One possible explanation for these various findings is that the association between age and the incidence of HCV is different for sexually transmitted and non-sexually transmitted HCV and that, when adjusting for behaviour and HIV treatment, that increasing age is in fact associated with a reduced incidence. Another explanation is that the age effect is different in countries or regions with different patterns of HCV epidemiology. In countries with rising HCV incidence or prevalence, sexual contacts of older MSM might have a higher HCV prevalence. This effect might be less or reversed in countries, like Australia, with a stable or falling HCV incidence or prevalence. However, an explanation for this association remains speculative.

On multivariate analysis the only HIV disease marker associated with a higher incidence of HCV was increased CD4 nadir, even though both virological suppression and a higher recent CD4 cell count were associated with an increased incidence of HCV in the univariate analysis. A Dutch case-case control study matching HCV-HIV co-infected MSM to HIV mono-infected individuals over the period 2009 to 2014 found an association between higher recent CD4 cell count and HCV risk and no association with CD4 nadir, but did not examine adjusted multivariate estimates [5]. This case control study, however, could not account for changes in HIV virological suppression and CD4 cell count which occurred over time. The MACS cohort observed a reduced HCV risk with higher recent CD4 cell count, but only in those with a CD4 nadir greater than 500, but again the population was not restricted to HIV positive MSM with no history of injecting drug use [7]. This suggests that degree of past immunosuppression does reduce HCV risk when adjusted for HIV virological suppression, degree of subsequent immune recovery and the biological and behavioural correlates of STIs. This also raises the possibility that the relationship between sexually transmitted HCV risk and antiretroviral therapy, recent CD4 cell count and CD4 cell count nadir might be different for early treated patients with high CD4 cell count nadir than for late treated patients with low CD4 cell count nadir. Possible mechanisms could include reduced inflammation or genital ulceration occurring in the previously severely immunosuppressed or a role for the immune response in sexual transmission of HCV other than passive transport of HCV across a damaged membrane.

We observed no change in overall HCV incidence over the study period. Studies in other countries have observed an increase in incidence of both sexually transmitted HCV and injecting transmitted HCV [21]. However, the increase in HCV transmission in the general community in Australia has not been observed over this period [23]. This suggests there may be an epidemiological link between HCV incidence in the general community (mostly driven by injecting drug use) and HCV incidence in HIV positive MSM with no history of injecting drug use. Overlapping of risk populations would be one such plausible link. A further implication of this would be that successful reduction of community HCV prevalence and incidence through HCV treatment as prevention could translate into reduced sexually transmitted HCV incidence in HIV positive MSM.

We did, however, observe a dramatic increase in syphilis incidence over the study period. Given the increased incidence of HCV was associated with incident syphilis, this could be expected to have an effect at the population level. Syphilis incidence increased more than three times during the study period, although in our study only 12 of 37 incident HCV cases were temporally associated with incident syphilis. Syphilis in our study population and in HIV positive MSM population in Australia is continuing to rise [23]. This can be expected to offset any reduction in HCV incidence gained through reducing prevalence as a result of treatment as prevention.

### Limitations

Our study is subject to certain potential limitations. First, sexual behaviour per se was not routinely collected in the course of clinical care at this specialist HIV clinic. We were therefore unable to adjust for this in our analysis. For this study, we chose anorectal chlamydia as a marker of sexual behaviour but not increased biological risk and syphilis as a marker of both. The parallel rise in chlamydia and syphilis incidence suggests that they are both linked to sexual behaviour.

Secondly, although anorectal chlamydia is not usually associated with genital ulcerations, some cases are associated with lymphogranuloma venereum chlamydia subtypes which can be associated with ulceration. At our centre, less than 2% of anorectal chlamydia is associated with LGV serovars [24]. Similarly not all cases of incident syphilis are necessarily associated with anogenital ulceration. We did not exclude LGV from cases of anorectal chlamydia or syphilis without apparent ulceration, which if anything would be likely to have increased the syphilis HCV risk association and decrease the anorectal chlamydia HCV risk association.

Thirdly, there may have been under-reporting or under-recording of injecting drug use at the time at which HIV acquisition risk factors are recorded, or patients may have commenced or ceased injecting drug use at a later time. To minimise the bias of misclassification of injecting drug use we additionally examined the clinical record of patients diagnosed with HCV infection and excluded two patients who had not previously reported injecting drug use but did so at the time of HCV diagnosis. The possibility of misclassification of the remaining patients remains although virtually all clinicians ask about this specific risk factor with every HCV infection.

Fourthly, patients included in this study had a high CD4 cell count nadir. From 2009 to 2014 the CD4 cell count threshold for intiation of ART was 500 cells/uL and from 2014 all patients were recommended to commence ART. Therefore most newly diagnosed patients had short periods of monitoring without ART. Our study controlled for delayed or early treatment initiation by including virological suppression and CD4 cell count nadir as covariates in the multivariate analysis. Previous studies performed at an earlier time in the HIV epidemic may have patients with much more advanced HIV infection and greater periods of observation prior to virological suppression. Accordingly these data may not be applicable to HCV transmission in resource limited settings or in older cohorts where HIV patients often have lower CD4 counts and may have an immunological susceptibility to HCV acquisition.

Fifth, very few patients were identified with possible incident HBV infection in our cohort. Our previous work has shown a low incidence of HBV infection in HIV negative MSM of 0.2 per 100 person years [25]. Our HIV positive population has much higher levels of vaccination than their HIV negative peers due to an active vaccination program. Furthermore lamivudine, tenofovir and emtracitabine, the most commonly used antiretroviral drugs, are known to have a powerful protective effect against HBV infection [26]. Cox regression analysis was not performed on cases of possible incident HBV infection. Our study showed a high rate of prior HBV exposure with 28% of MSM having a recorded positive HBV core antibody. The Australian HIV Observational Database, a prospective cohort of people living with HIV which includes approximately 80% men who have sex with men reports that 44.1% of tested individuals are HBV core antibody positive [27].

Finally, our calculation of the at-risk periods around incident syphilis or chlamydia was greater than the ‘actual’ period over which these STIs were present. We did this to take account of the fact that hepatitis C may have a prolonged period of sero-conversion but by doing this we acknowledge that actual hazard ratio is likely to be underestimated because the denominator period is longer. Thus it is likely that the true hazard ratio for syphilis is actually higher than we found and also it is possible that chlamydia has some small increased risk of HCV associated with it. However undertaking a formal prospective cohort study with frequent STI measures to limit this would be very expensive given the relatively low incidence of hepatitis C.

## Conclusions

Hepatitis C virus infection in HIV positive MSM is associated with syphilis but not anorectal chlamydia which suggests a biological rather than behavioural risk modification. This suggests that the potential breach to the genital or anorectal mucosa is associated with HCV risk rather than condomless receptive anal sex per se. Rising syphilis incidence will increase the periods during which the individual is a increased risk of HCV acquisition. This may offset declines in HCV transmission through HCV treatment as prevention.

## Abbreviations

aHR: Adjusted hazards ratio
AIDS: Acquired immune deficiency syndrome
ART: Antiretroviral therapy
CPMS: Clinical patient management system
DNA: Deoxyribonucleic acid
EIA: Enzyme-linked immunosorbent assay
HBV: Hepatitis B virus
HCV: Hepatitis C virus
HIV: Human immunodeficiency virus
IDU: Injecting drug use
LGV: Lymphogranuloma venereum
MACS: Multicentre AIDS Cohort
MDU PHL: Melbourne Diagnostic Unit Public Health Laboratory, University of Melbourne
MSHC: Melbourne sexual health centre
MSM: Men who have sex with men
NAAT: Nucleic acid amplification test
PCR: Polymerase chain reaction
PY: Person years
RNA: Ribonucleic acid
RPR: Rapid plasma reagin
SD: Standard deviation
STI: Sexually transmitted infection
TPPA: Treponema pallidum particle agglutination assay
VIDRL: Victorian Infectious Diseases Reference Laboratory
